# Clinical and biochemical footprints of inherited metabolic diseases. XV. Epilepsies

**DOI:** 10.1016/j.ymgme.2023.107690

**Published:** 2023-08-26

**Authors:** Itay Tokatly Latzer, Nenad Blau, Carlos R. Ferreira, Phillip L. Pearl

**Affiliations:** a Department of Neurology, Boston Children’s Hospital, Harvard Medical School, Boston, MA, USA; b Sackler Faculty of Medicine, Tel-Aviv University, Tel Aviv, Israel; c Division of Metabolism, University Children’s Hospital, Zürich, Switzerland; d National Human Genome Research Institute, National Institutes of Health, Bethesda, MD, USA

**Keywords:** Seizures, Epilepsy, Metabolic, Neurometabolic, Diagnosis, Treatment

## Abstract

We provide a comprehensive overview of inherited metabolic disorders (IMDs) in which epilepsy is a prominent manifestation. Our unique database search has identified 256 IMDs associated with various types of epilepsies, which we classified according to the classic pathophysiology-based classification of IMDs, and according to selected seizure-related factors (neonatal seizures, infantile spasms, myoclonic seizures, and characteristic EEG patterns) and treatability for the underlying metabolic defect. Our findings indicate that inherited metabolic epilepsies are more likely to present in the neonatal period, with infantile spasms or myoclonic seizures. Additionally, the ~20% of treatable inherited metabolic epilepsies found by our search were mainly associated with the IMD groups of “cofactor and mineral metabolism” and “Intermediary nutrient metabolism.” The information provided by this study, including a comprehensive list of IMDs with epilepsy stratified according to age of onset, and seizure type and characteristics, along with an overview of the key clinical features and proposed diagnostic and therapeutic approaches, may benefit any epileptologist and healthcare provider caring for individuals with metabolic conditions.

## 1. Introduction

The “Metabolic Footprints Series” aims to present a comprehensive list of inherited metabolic disorders (IMDs) associated with specific medical conditions. Thus far, articles published under the umbrella of this series covered the involvement of IMDs with movement disorders, liver diseases, cardiovascular diseases, psychiatric presentations, cerebral palsy phenotypes, dermatoses, gastrointestinal symptoms, myopathies, neoplasias, ocular phenotypes, kidney, and ear diseases. This review, the 15th of this series, is dedicated to inherited metabolic epilepsies (IMEs), IMDs in which the metabolic impairment leads to epilepsy being one of their prominent symptoms. We first discuss the metabolic pathomechanisms leading to seizures and epileptogenesis, along with the classification of IMEs. This is followed by descriptions of the clinical presentation, diagnostic approach, and management of IMEs.

## 2. Materials and methods

Source of the information was IEMbase the knowledgebase of IMDs (http://www.iembase.org) [1]. As of June 9, 2023, IEMbase tabulates 1878 IMDs and 4107 corresponding clinical and biochemical signs and symptoms grouped in 21 organ systems and conditions. Clinical symptoms associated with epilepsies (*n* = 50) were extracted from the ‘Neurologic’ group.

The nosology of IMDs [2] was reclassified according to the International Classification of Inherited Metabolic Disorders, ICIMD [3].

### 2.1. Classification of Inherited Metabolic Epilepsies

#### 2.1.1. Classification based on metabolic pathomechanism

A wide range of metabolic pathomechanisms can lead to seizures and epileptogenesis, serving as the basis by which IMEs are classified [4]. Seizures may result from the neurotoxic accumulation of low-molecular-weight compounds (in organic acidemias, aminoacidemias, and urea cycle disorders, the “Small Molecule Disorders”), a deficiency of low-molecular-weight compounds (in asparagine synthetase deficiency or serine biosynthetic disorders), or improperly synthesized or recycled poorly diffusible larger compounds (in peroxisomal disorders, lysosomal storage disease, and congenital disorders of glycosylation, the “Complex Molecule Disorders”). Seizures may also develop from a disturbed generation of cellular energy (in mitochondrial diseases, GLUT-1 deficiency, and fatty acid oxidation disorders, the “Disorders of Energy Metabolism”) and deficient cofactors, minerals, and vitamins (such as in pyridoxine-dependent epilepsy, cerebral folate deficiency, or biotinidase deficiency). Metabolic alterations leading to direct disruption of neurotransmission and cell signaling processes (such as in disorders of γ-aminobutyric acid [GABA] catabolism) affect the excitatory:inhibitory balance of the brain, potentially leading to epileptogenesis and development of seizures [5].

#### 2.1.2. Classification based on seizure and epilepsy characteristics

**Fig. 1.**
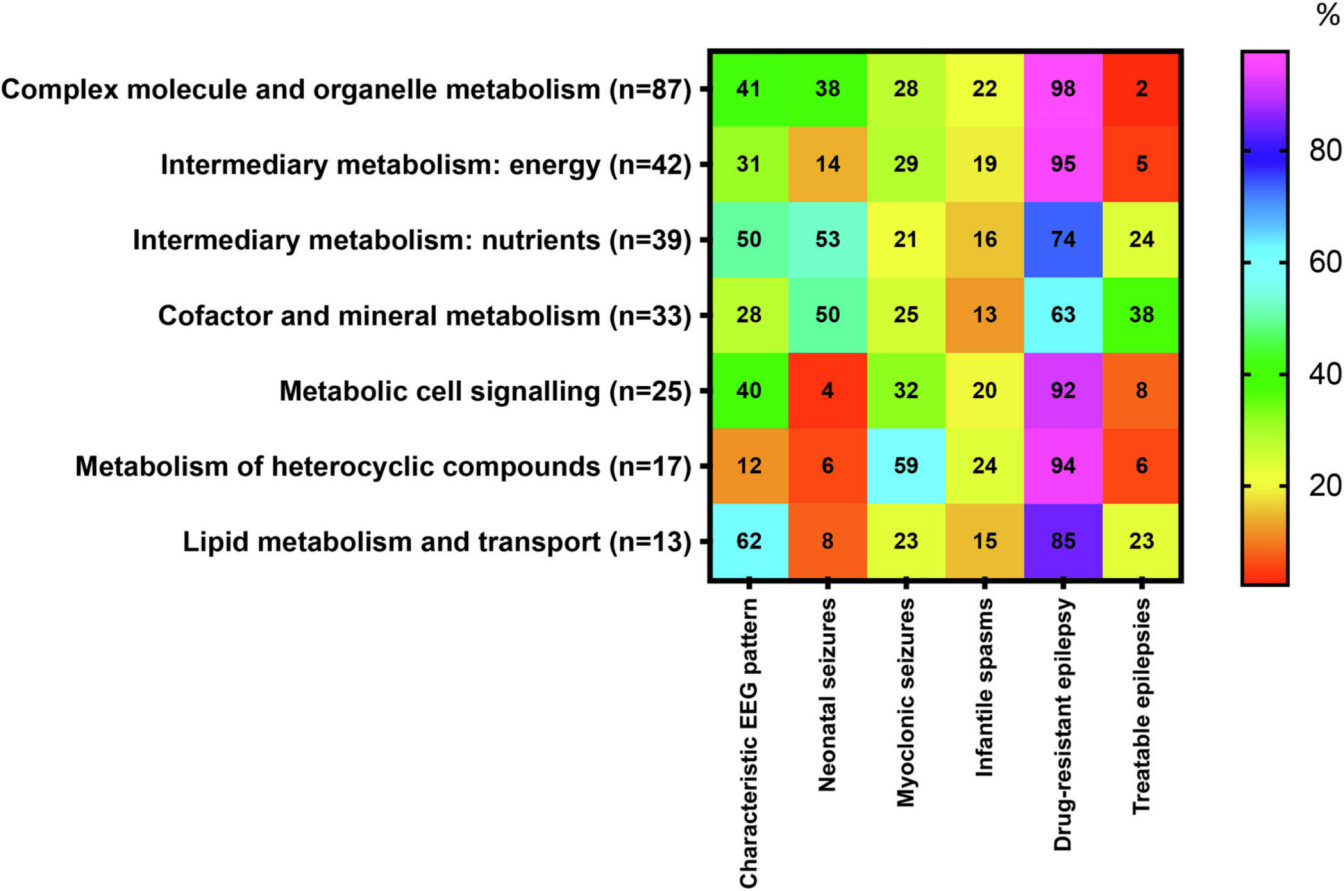
Occurrence (%) of symptoms associated with disorders presenting with epilepsies in seven categories of IMDs (according to ICIMD). The percentages for epilepsies or treatability were calculated using as the denominator the total number of IMDs in each category presenting with any seizure or epilepsy characteristic. The heat scale ranges from red (0%) for diseases with no particular reported symptoms to violet (100%) for diseases with particular symptoms occurring more frequently within the disorders group. For the definition of six categories of seizure or epilepsy characteristics, see Supplemental Table S1. For interpretation of the references to color in this figure legend, the reader is referred to the web version of this article.

The search conducted as part of this study identified 256 IMEs. In addition to presenting these identified IMEs according to the classical pathophysiology-based classification, we presented them according to six groups representing their important seizure and epilepsy-related distinguishing characteristics. IMEs presenting in the neonatal period are most commonly associated with myoclonic seizures. However, they may also present with electrographic seizures or other electroclinical seizure types typical for the neonatal period, specifically clonic, tonic, automatisms, epileptic spasms, autonomic, and behavioral arrests [6]. Seizures presenting in the neonatal period require specific consideration since there is a higher likelihood of their etiology stemming from a metabolic origin. Notorious examples are Early Infantile Developmental and Epileptic Encephalopathy (EIDEE) [7], Infantile Epileptic Spasms Syndrome [8], and Progressive Myoclonus Epilepsies (PME) [9]. Depending on the degree of metabolic impairment, other IMEs may present at different ages, with different seizure types (in certain instances mimicking generalized idiopathic epilepsies), and with different severities. According to the search conducted in this study, neonatal seizures characterized 78/256 (30%) of the IMEs. This strengthens the studies and guidelines suggesting metabolic etiologies are common for neonatal seizures [10]. Therefore, when neonatal seizures without a known etiology are encountered, a metabolic investigation is advised [6]. Our findings indicated neonatal seizures had the highest association with the IMD groups of “Intermediary metabolism” (53%) and “cofactor and mineral metabolism” (50%). This information stresses the importance of pursuing a metabolic workup especially if other manifestations indicating these groups of IMDs are present. Myoclonic seizures, which were notable in 73/256 (29%) of the IMEs, emphasize the importance of recognizing these types of seizures in relation to a possible metabolic etiology. Myoclonic seizures can be seen in various IMEs, such as pyridoxine-dependent and pyridoxal phosphate-responsive epilepsies, mitochondrial diseases, glycine encephalopathy, and the progressive myoclonus epilepsies (neuronal ceroid lipofuscinoses, myoclonic epilepsy with ragged red fibers [MERRF], and Gaucher disease type III) [11]. As also indicated by our results, myoclonic seizures were noted to occur in higher association in the groups “Metabolism of heterocyclic compounds” (59%), “Metabolic cell signaling” (32%), and “Intermediary metabolism: energy” (29%). Interestingly, infantile spasms, listed as part of 48/256 (19%) of the IMEs, did not have a specific predilection for any of the pathophysiology-based groups (Fig. 1). This resonates with reports from the National Infantile Spasms Consortium [12], describing that one of the main etiological groups of infantile spasms is metabolic; however, no specific metabolic condition surpasses the others in occurrence.

### 2.2. Clinical aspects of inherited metabolic epilepsies

#### 2.2.1. Presentation

IMEs manifesting in early versus late stages of life differ in their clinical presentation. In newborns and infants, epilepsy originating from a metabolic source can be accompanied by signs and symptoms including weakness, tone changes, vomiting, respiratory compromise, and encephalopathy [13]. IMEs such as organic and aminoacidurias, urea cycle disorders, and disorders of energy metabolism (e.g., mitochondrial diseases) may emerge following stressors such as fasting/protein or carbohydrate consumption, intercurrent illness, or medications [14,15]. Congenital glycosylation disorders, lysosomal storage disorders, or peroxisomal disorders may be accompanied by dysmorphic features, microcephaly, or other systemic signs such as hepatosplenomegaly and ophthalmologic symptoms [16–18]. Other presentations suggesting a metabolic origin in these younger age groups are movement disorders typical of diseases in which chemical neurotransmission is disrupted [19]. Notably, in the case of infants presenting with signs suspicious of nonaccidental injuries such as subdural and retinal hemorrhages, cobalamin C deficiency, glutaric acidemia type I, and Menkes disease should also be part of the differential [20]. Some of the disorders listed above can also present at first in older children, adolescents, and adults, reflecting gradually developing deficits resulting from lower degrees of metabolic impairments. Notable manifestations pointing to a metabolic etiology in these older ages include an acute or progressive appearance of motor or language regression and psychiatric symptoms (for example, in lysosomal storage diseases and mitochondrial diseases), movement disorders such as dystonia (in glutaric aciduria, Lesch-Nyhan syndrome, GM1 gangliosidosis, brain iron accumulation, and mitochondrial diseases), ataxia (milder cases of maple syrup urine disease and urea cycle disorders), stroke (in homocysteinemias, lactic acidosis and stroke-like episodes [MELAS]), and rhabdomyolysis (in carnitine and fatty acid oxidation disorders) [21]. The seizure-related characteristics of the most prominent IMEs are described in Table 1.

#### 2.2.2. Diagnostic approach

The challenge to diagnose IMEs is mainly based on their nonspecific clinical presentation, as described above. Therefore, a diagnostic metabolic investigation should be pursued in cases of unexplained epilepsy presenting early or later in life, especially if some of the symptoms listed above are present. Recently, genetic diagnostic methods, including next-generation sequencing (NGS) gene panels and exome sequencing (ES), have become more accessible. When applicable, these tests should be the first line in diagnosing metabolic disorders [22,23].

Chromosomal microarray analysis (CMA), methylation studies, and repeat expansion tests are less specific for IMEs but may also lead to the diagnosis in some instances [22]. Notably, tests assessing the mitochondrial DNA (mtDNA) genome should also be completed if the clinical picture suggests a mitochondrial disease [24]. Genetic tests have more diagnostic relevance than metabolic tests in subjects with seizures stemming from an undetermined etiology [25]. Nevertheless, basic plasma and urine-based metabolic tests that are typically accessible have several advantages: they may reveal the diagnosis of an IME faster than the genetic option (resulting in rapid commencement of treatments that could be life-saving), complement the interpretation of genetic “variants of uncertain significance,” and determine the progressive degree of an IME that has been diagnosed [26]. A step-wise and updated diagnostic approach to IMEs, including many of the standard tests listed in Supplemental Table S1, along with CSF and neuroimaging studies (magnetic resonance imaging and spectroscopy), has been recently described [27].

**Fig. 2.**
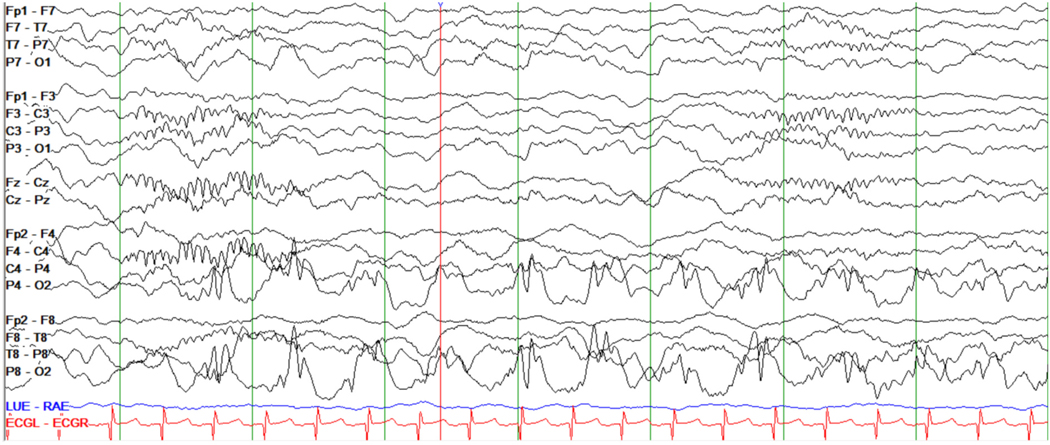
EEG tracing showing rhythmic high amplitude delta with (poly)spikes (RHADS) in an infant with drug-resistant epilepsy secondary to *POLG1*-related mitochondrial disease.

**Fig. 3.**
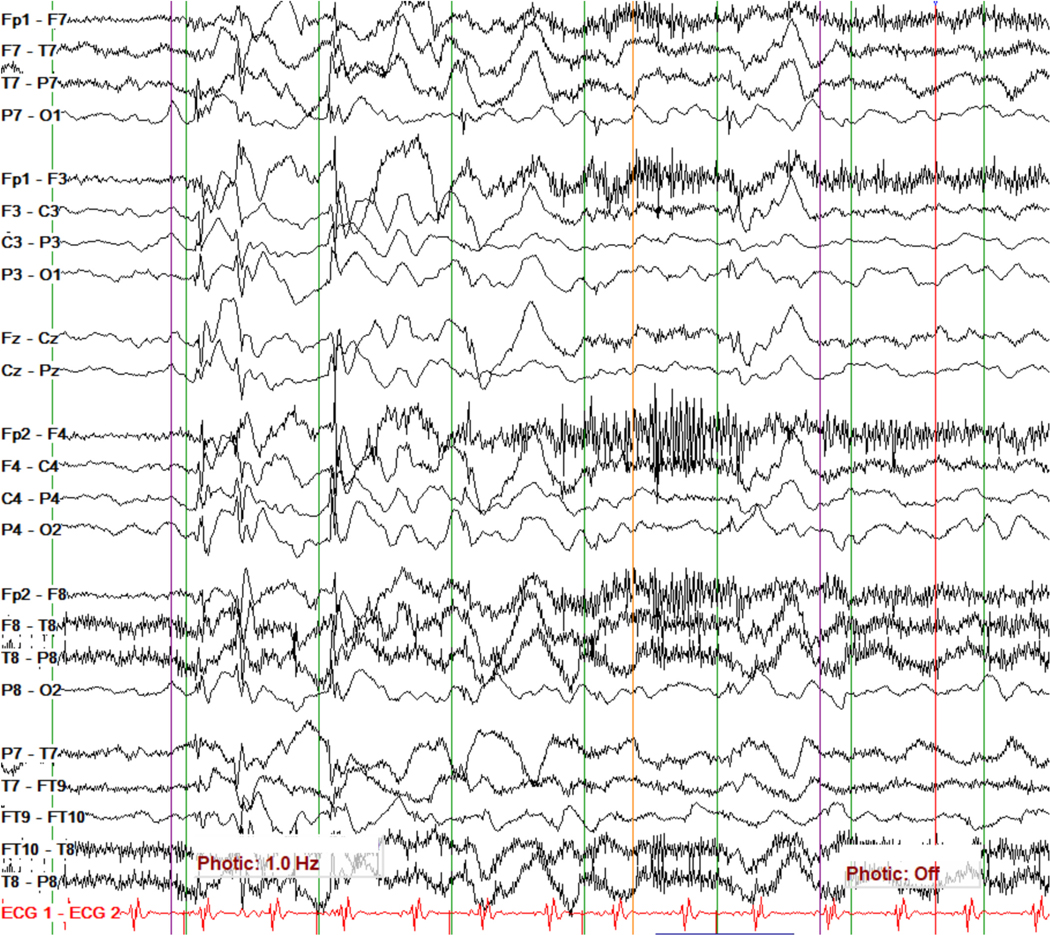
EEG tracing showing a marked response to low-frequency (1 Hz) photic stimulation in a toddler with pathogenic variants in *TPP1* and a diagnosis of neuronal ceroid lipofuscinosis type 2 (CLN2).

The electroencephalogram (EEG) is not specific but highly sensitive for certain IMEs and therefore offers clinical utility in diagnosing these conditions. EEG tracing patterns that may distinguish particular IMEs include repetitive high amplitude delta with spikes/polyspikes (RHADS) in polymerase gamma (POLG)-related mitochondrial disease (Fig. 2) [28,29], comb-like rhythm with 7- to 9-Hz central activity in patients with maple syrup urine disease (MSUD) [30] and propionic acidemia [31], marked photosensitivity in neuronal ceroid lipofuscinoses (Fig. 3) [32], giant somatosensory evoked potentials in the progressive myoclonic epilepsies [33], or central spikes in Tay-Sachs disease and biotinidase deficiency [34,35]. Other EEG patterns are also associated with various non-metabolic conditions but are seen at higher rates with metabolic ones. These include a burst-suppression pattern seen in patients with glycine encephalopathy/nonketotic hyperglycinemia (GE/ NKH), untreated classic phenylketonuria (PKU), neonatal citrullinemia, MSUD, molybdenum cofactor/sulfite oxidase deficiency, and holocarboxylase synthetase deficiency, and hypsarrhythmia seen in peroxisomal biogenesis disorders, neuroaxonal dystrophy, GE/NKH, PKU, congenital defects of glycosylation, and Menkes disease [11,31,36–39]. This information is supported by findings from this study’s search, showing that characteristic EEG patterns were present in 97/256 (38%) of the conditions listed, with hypsarrhythmia reported in 35% and a burst-suppression pattern in 15%, and with higher occurrence rates in the IMD groups of “Lipid metabolism and transport” (57%) and “Intermediary metabolism: nutrients” (51%) (Fig. 1 and Supplemental Table S1).

#### 2.2.3. Treatment

Approximately 20% of the IMEs described in this study were determined as “treatable” (Table 2). We considered an IME as treatable only if a targeted treatment prevents or improves the seizures (and other manifestations) by addressing the specific metabolic defect (in contrast to standard antiseizure medicines). The remaining conditions (~ 80%) were regarded as drug-resistant. These findings are not surprising, as the currently available therapies for most IMEs are supportive only [40,41]. However, outcomes of the “Treatable” IMEs could be substantially improved with targeted treatment [40] and thus should be highlighted. This study’s search shows an overall low occurrence rate of treatable IMEs in all the groups listed (Fig. 1). This is especially true for conditions listed under the groups “Metabolism of heterocyclic compounds” (0%) and “Complex molecule and organelle metabolism” (2%). However, it is also noticeable that 38% of the disorders listed in the “Cofactor and mineral metabolism” group and 24% of those listed under “Intermediary metabolism: nutrients” do have treatments. These findings are supportive of evidence showing that pyridoxine-dependent epilepsy and other vitamin-responsive diseases (e.g., cerebral folate deficiency) may be corrected by early administration of the particular vitamin or cofactor [39,42–45], and that treatment of seizures that originate from electrolyte disturbances or hypoglycemia may be terminated with the appropriate replacement therapy [11,40]. The exclusion of hypoglycemia should be further emphasized in the context of congenital hyperinsulinism causing refractory hypoglycemia that may lead to seizures. It is also important to mention the treatability of carbamoyl phosphate synthetase, aspartate transcarbamylase, and dihydroorotase (CAD) deficiency by supplementation of uridine [46]. Other therapeutic approaches that reflect our findings include the dietary treatments that may benefit Glut-1 deficiency syndrome (ketogenic diet) [47], phenylketonuria (low-phenylalanine diet) [48], urea cycle disorders (protein restriction) [49], fatty acid oxidation disorders (fat restriction) [50], and some organic and amino acidurias (certain protein restrictions) [51]. Importantly, in the last decade, there have been dramatic advancements in developing gene and enzyme replacement therapies for metabolic disorders [52]. Notable examples are enzyme replacement therapies for Gaucher disease [53] and tripeptidyl-peptidase 1 deficiency (CLN2 disease) [54], and gene replacement therapies for X-linked adrenoleukodystrophy [55,56] and aromatic L-amino acid decarboxylase (AADC) deficiency [57]. Many other gene and enzyme replacement therapies are currently being investigated by preclinical and clinical trials [40]. Lastly, it is crucial to be aware that while certain typical anti-seizure medications may lessen the seizure frequency and intensity in some IMEs, they may worsen seizures in others. Pronounced examples include valproate, which may worsen seizures in patients with mitochondrial defects, urea cycle disorders, fatty acid oxidation disorders, and Glut-1 deficiency syndrome; a ketogenic diet that may intensify the seizures in subjects with pyruvate carboxylase deficiency and organic acidurias; and phenytoin that may increase seizures in Unverricht–Lundborg disease [26,27]. Finally, while it is conceivable that individuals with succinic semialdehyde dehydrogenase deficiency (SSADHD) may benefit from vigabatrin since it is an irreversible inhibitor of GABA transaminase, it has not shown consistent or sustained benefit [58].

## 3. Conclusions

In this study, we provide a comprehensive overview of inherited metabolic disorders in which epilepsy is one of the major manifestations. While the occurrence rate of each of the 256 individual conditions identified by this study is rare, their cumulative prevalence is significant. Additionally, as revealed by our findings, the high likelihood of IMEs presenting in the neonatal period, with infantile spasms or myoclonic seizures, prompts investigating a metabolic etiology in applicable clinical scenarios. As also seen by our search, the fact that there exists effective and targeted therapy for ~20% of the IMEs emphasizes the importance of their clinical recognition. This information, accompanied by an overview of their key clinical characteristics and proposed diagnostic and therapeutic approaches to IMEs, may benefit any epileptologist and healthcare provider caring for individuals with metabolic conditions. This study represents the 15th issue in a series of scholarly summaries providing an inclusive and up-to-date list of inherited metabolic disorders associated with epilepsy. The complete list can be accessed at www.iembase.org/gamuts and will be curated and updated regularly.

## Supplementary Material

1

## Figures and Tables

**Table 1:** Seizure-related characteristics of the most prominent inherited metabolic epilepsies.

Category	Inherited metabolic epilepsy	Age of seizure onset	Predominant seizure types	EEG pattern

**Small Molecule Disorders**	Phenylketonuria (untreated)	Neonatal period to Infancy	GTCs; myoclonic; epileptic spasms	Hypsarrhythmia; epileptic spasms; diffuse background slowing, focal sharp waves; irregular generalized spike and slow waves
	Maple syrup urine disease	Neonatal period to early childhood (type dependent)	Myoclonic; GTCs	Diffuse slowing; loss of reactivity to auditory stimuli; bursts of a central mu-like rhythm termed “comb-like rhythm.”
	Homocystinuria	Infancy to early childhood (type dependent)	GTCs; focal; epileptic spasms	Focal interictal epileptiform discharges; diffuse background slowing
	Isovaleric acidemia	Neonatal period	Tonic: focal motor	Burst suppression; dysmature sleep features
	Propionic acidemia	Neonatal period and infancy	Myoclonic; focal; atypical absence	Background disorganization; marked frontotemporal and occipital slow-wave activity
	Methylmalonic acidemia	Neonatal period and infancy	Diffuse tonic; focal with bilateralization; eyelid clonus with simultaneous upward eye deviation	Multifocal spikes; depressed background; excessive generalized slowing; lack of sleepspindles; hypsarrhythmia
	3-Methylglutaconic Aciduria	Neonatal period and infancy	Epileptic spasms; GTCs	multifocal spike-wave discharges
	Glutaric Acidemia Type I	Infancy to early childhood (precipitated by metabolic decompensation or illness).	Epileptic spasms	Background slowing; generalized spike-and-wave and mixed multifocal discharges
	Urea Cycle Disorders	Neonatal period to late childhood (disorder dependent)	Subclinical electrographic seizures; generalized; focal motor	Low amplitude diffuse slowing; multifocal epileptiform discharges; monorhythmic theta activity; burst-suppression (citrullinemia)
**Large Molecule Disorders**	Congenital Disorders of Glycosylation	Infancy to early childhood (disorder dependent)	Focal; atonic, myoclonic; epileptic spasms; migrating focal	Hypsarrhythmia
	Neuronal ceroid lipofuscinoses	Early childhood, adolescence, and adulthood (disorder dependent)	Myoclonic; atonic; multifocal; GTCs; behavioral arrest	Early attenuation and progressive loss of background (vanishing EEG); occipital spikes precipitated by low-frequency photic stimulation; giant somatosensory-evoked potentials
	Tay–Sachs and Sandhoff Diseases	Late childhood to adolescence	Focal motor: atypical absence	Normal early in the disease course; With disease progression- background activity slows and bursts of high amplitude delta activity and very fast central spikes appear; diffuse spike and sharp waves noted with acoustically induced myoclonic seizures; amplitude decreases in later stages
	Krabbe Disease (Globoid Cell Leukodystrophy)	Late childhood to adolescence	Focal motor; GTCs; epileptic spasms	Hypsarrhythmia-like pattern- irregular slow activity and multifocal low-amplitude discharges; independent posterior-temporal and central β activity superimposed over slow high-amplitude waves; diffuse attenuation in later stages
	GM1 Gangliosidosis Types I and II	Late infancy to early childhood	Myoclonic; GTCs	Diffuse irregular slow activity; in type II-fluctuating 4–5 Hz temporal rhythmic discharges
	Metachromatic Leukodystrophy	Early to late childhood	Focal	Diffuse background slowing; focal or multifocal epileptiform discharges
	Gaucher Disease Type III	Infancy to early childhood	GTCs; myoclonic	Diffuse polyspike discharges with occipital predominance; rhythmic runs of 6–10 Hz spikes or sharp waves
	Peroxisomal Biogenesis Disorders	Neonatal period to early infancy	Focal; myoclonic; atypical flexor spasms; GTCs (rarely)	Hypsarrhythmia; burst suppression; multifocal epileptiform activity
**Disorders of Energy Metabolism**	Pyruvate Dehydrogenase Deficiency	Neonatal period to early infancy	Myoclonic; epileptic spasms	Multifocal slow spike-wave discharges; hypsarrhythmia
	Pyruvate Carboxylase Deficiency	Neonatal period to early infancy	Epileptic spasms	Hypsarrhythmia
	Leigh Syndrome	Early childhood to adolescence	Epileptic spasms; epilepsia partialis continua (continuous focal seizures)	Nonspecific; multifocal slow spike-wave discharges; hypsarrhythmia
	mtDNA Depletion Syndromes	Late childhood to adolescence	Epilepsia partialis continua (continuous focal seizures); myoclonic; GTCs	Rhythmic high amplitude delta with (poly) spikes in POLG-1 associated hepatocerebral degeneration).
	MERRF and MELAS	Late childhood to adolescence	Myoclonic; focal motor; epilepsia partialis continua (in MELAS)	
	Glut-1 Deficiency	Neonatal period, early infancy, and may appear later.	Multifocal, atonic, typical and atypical absence, GTCs, myoclonic	Can be normal interictally, focal, or generalized slowing or attenuation; generalized, focal, or multifocal 2.5–4 Hz spike-and-wave discharges
	Creatine synthesis defects	Late infancy to early childhood	GTCs; focal	Background slowing; generalized spike-and-wave, polyspike, or multifocal epileptiform discharges
**Disorders of Cofactors, Minerals and Vitamins**	Pyridoxine-dependent epilepsy, folinic acid-responsive epilepsy, pyridoxal-L-phosphate-responsive epilepsy	Neonatal period to early infancy; milder variants later in childhood	Epileptic spasms; focal motor; myoclonic; atonic	Initially-generalized bursts of high amplitude delta waves interspersed with spike and sharp waves and interburst intervals of asynchronous attenuation; after treatment- conversion to classic burst-suppression; later-normalization
	Molybdenum Cofactor Deficiency and Sulfite Oxidase Deficiency	Neonatal period to early infancy	Focal motor; myoclonic; GTCs	Burst-suppression pattern; multifocal spike-wave discharges
	Cerebral Folate Deficiency	Infancy to late childhood (disorder dependent)	Myoclonic; GTCs	Multifocal epileptiform activity; diffuse background slowing
	Methylenetetrahydrofolate Reductase Deficiency	Infancy	Epileptic spasms; myoclonic; GTCs	Diffuse background slowing; continuous spike-wave complexes or multifocal spikes
	Early-Onset Multiple Carboxylase Deficiency (Holocarboxylase Synthetase Deficiency)	Neonatal period to early infancy	GTCs; focal motor; multifocal myoclonic	Burst-suppression pattern; multifocal epileptiform activity
	Biotinidase Deficiency	Neonatal period to early infancy	Epileptic spasms; GTCs; focal motor; myoclonic	Burst suppression; poorly organized and; slow awake background; lack of typical sleep elements; frequent spikes and spike-slow-wave discharges
	Menkes Disease (Copper Transport Disease)	Infancy to early childhood	Focal motor; epileptic spasms; stimulation-induced myoclonic jerks	Multifocal spike and slow-wave activity (hypsarrhythmia-like); burst suppression
**Disorders of Neurotransmitters**	Aromatic L-amino acid decarboxylase deficiency	Infancy to early childhood	Focal or generalized (relatively rare)	Nonspecific
	Glycine encephalopathy	Neonatal period to early infancy	Myoclonic; epileptic spasms	Hypsarrhythmia; burst-suppression; multifocal epileptiform activity
	Serine Biosynthesis Defects	Neonatal period to early infancy	Epileptic spasms; myoclonic	Hypsarrhythmia; burst-suppression; multifocal spikes and sharp waves
	Succinic Semialdehyde Dehydrogenase Deficiency	Late childhood, but may onset from infancy to adulthood	Focal motor; absence; myoclonic	Diffuse background slowing; focal and multifocal interictal epileptiform activity

EEG-Electroencephalography GTCs-Generalized tonic-clonic MELAS-Mitochondrial Encephalopathy, Lactic Acidosis, and Stroke-like episodes MERRF-Myoclonic epilepsy with Ragged Red Fibers.

**Table 2:** Treatable inherited metabolic epilepsies described in this study.

Disorder	Gene	Treatment	Developing/Established Novel Treatment (ClinicalTrials.gov)

Carbamoyl phosphate synthetase, aspartate transcarbamylase, dihydroorotase (CAD) deficiency	*CAD*	Uridine	
Carbamoyl phosphate synthetase I deficiency	*CPS1*	Protein restriction, hydration, citrulline and arginine supplementation, ammonia scavengers, and hemodialysis.	
Ornithine transcarbamylase deficiency	*OTC*	Protein restriction, hydration, arginine supplementation, ammonia scavengers, and hemodialysis.	mRNA-based ERT (preclinical trials) NCT03767270; Gene replacement therapy (clinical trials) NCT02991144
Arginase 1 deficiency	*ARG1*	Protein restriction, hydration, ammonia scavengers, and hemodialysis.	ERT-Pegzilarginase (clinical trials) NCT03378531
Cystathionine beta-synthase deficiency	*CBS*	Protein-specific based diet, methionine restriction; pyridoxine, betaine	ERT-Pegtibatinase NCT03406611
Nonketotic hyperglycinemia due to glycine decarboxylase deficiency	*GLDC*	Sodium benzoate, NMDA antagonists	
Phosphoserine aminotransferase deficiency	*PSAT1*	Serine and glycine supplementation	
Phosphoglycerate dehydrogenase deficiency	*PHGDH*	Serine and glycine supplementation	
Phosphoserine phosphatase deficiency	*PSPH*	Serine supplementation	
Glucose transporter 1 deficiency	*SLC2A1*	Ketogenic diet	
3-Hydroxy-3-methylglutaryl-CoA synthase deficiency	*HMGCS2*	Avoidance of long periods of fasting, and a low-fat diet	
3-Hydroxy-3-methylglutaryl-CoA lyase deficiency	*HMGCL*	Long-term protein and fat restriction; L-carnitine supplementation	
Arginine:glycine amidinotransferase deficiency	*GATM*	Creatine	
Guanidinoacetate methyltransferase deficiency	*GAMT*	Creatine	
X-linked adrenoleukodystrophy and adrenomyeloneuropathy	*ABCD1*	Gene therapy-Elivaldogene autotemcel (Lenti-DTM, SKYSONATM).	Ex Vivo Gene Therapy with Lenti-DTM Lentiviral Vector NCT01896102
Phenylalanine hydroxylase deficiency	*PAH*	Low-phenylalanine and low-protein diet; cofactor tetrahydrobiopterin (Kuvan); EST with pegvaliase (Palynziq)	mRNA-based gene therapy (preclinical trials) NCT04480567; Gene-replacement therapy (clinical trials) NCT03952156
Sterol 27-hydroxylase deficiency	*CYP27A1*	Chenodeoxycholic acid	
PMM2-CDG	*PMM2*	Mannose supplementation (in a subgroup of patients); Acetazolamide	
Tripeptidyl-peptidase 1 deficiency	*TPP1*	ERT-Cerliponase alfa	NCT02485899, NCT04476862
6-Pyruvoyl-tetrahydropterin synthase deficiency	*PTS*	Phenylalanine-reduced diet; folinic acid supplementation; sapropterin dihydro-chloride; 5-hydroxytryptophan; L-dopa+carbidopa	
Pyridox(*am*)ine 5′-phosphate oxidase deficiency	*PNPO*	Pyridoxal 5′-phosphate	
Pyridoxal 5′-phosphate binding protein deficiency	*PLPBP*	Pyridoxine or pyridoxal 5′-phosphate	
Hyperprolinemia, type II	*ALDH4A1*	Pyridoxine	
Hypophosphatasia	*ALPL*	ERT-Asfotase alfa	NCT01203826, NCT02456038
Alpha-amino adipic semialdehyde (AASA) dehydrogenase deficiency	*ALDH7A1*	Pyridoxine; arginine supplementation; lysine restriction	
Biotinidase deficiency	*BTD*	Biotin	
Proton-coupled folate transporter deficiency	*SLC46A1*	Parenteral folate	
Folate receptor alpha deficiency	*FOLR1*	Folinic acid	
5,10-methylenetetrahydrofolate reductase deficiency	*MTHFR*	Betaine, hydroxocobalamin, and folate (in particular forms)	
Dihydrofolate reductase deficiency	*DHFR*	Folinic acid	
5,10-Methenyltetrahydrofolate synthetase deficiency	*MTHFS*	L-5-methyltetrahydrofolate and intramuscular methylcobalamin	
Vitamin D 1-α-hydroxylase deficiency	*CYP27B1*	Vitamin D	
Aromatic L-amino acid decarboxylase (AADC) deficiency	*DDC*	AAV-mediated gene transfer (intraputaminaly)-Eladocagene exuparvovec (Upstaza)	
Tyrosine hydroxylase deficiency	*TH*	L-dopa+carbidopa	

ERT: enzyme replacement therapy.

## Data Availability

Data will be made available on request.
